# Anti-VEGF and Beyond

**DOI:** 10.1155/2017/5815021

**Published:** 2017-10-31

**Authors:** T. Bertelmann, H. Kaymak, F. T. A. Kretz, M. J. Koss

**Affiliations:** ^1^Department of Ophthalmology, University Medical Center Göttingen, Göttingen, Germany; ^2^Artemis Eye Clinic, Dillenburg, Germany; ^3^Internationale Innovative Augenchirurgie, Duesseldorf, Germany; ^4^International Vision Correction Research Centre Network (IVCRC.net), Department of Ophthalmology, University of Heidelberg and Eyeclinics Ahaus-Raesfeld-Rheine, Dr. Gerl & Colleagues, Ahaus Lag, Germany; ^5^Department of Ophthalmology, Ruprecht-Karls-University Heidelberg and Augenzentrum Nymphenburger Höfe/Augenklinik Herzog Carl Theodor, Munich, Germany

Almost one decade ago, the first anti-VEGF drugs to treat neovascular eye disorders were approved by the respective health authorities and brought into routine clinical care. This “revolution” in the field of ophthalmology offered the treating physicians the chance to sustainably save vision for patients affected for the first time. Since these days, the understanding of the efficacy and safety profiles of the anti-VEGF drugs available has constantly grown. However, many open questions remain. Furthermore, new alternative and/or additional substances targeting VEGF and other factors such as PDGF and PIGF are on the horizon to alter and further improve our treatment strategies for neovascular eye diseases. This special issue focused on both topics, and many research groups shared their valuable research results to better understand the mode of action of various anti-VEGF as well as of new molecules to counteract neovascular eye diseases.

Illuminating the traditional indications, neovascular age-related macular degeneration (nAMD), diabetic macular edema (DME), and macular edema due to retinal vein occlusions (RVO), the questions advancing into the focus of interest for some time have been when and how to switch or switch back different anti-VEGF drugs, if at all switching these substances is favorable in comparison to maintaining the anti-VEGF in use to ensure the best clinical and anatomical outcome for the individual patient. In this respect, T. H. C. Tran et al. investigated the two-year outcome of Aflibercept in the treatment of pigment epithelium detachments (PED) refractory to Ranibizumab. They could show that in the short term, this switch was successful, whereas in the long term, no significant improvements were obtained and thus switching in this scenario appears to be questionable. A. Herbaut et al. share their results of eyes suffering from DME which were switched from Ranibizumab and/or Dexamethasone treatment to intravitreal Aflibercept injections. Their results show a significant functional and anatomical improvement after switching in the short term after 6 months. It would be interesting to experience, if these differences persist in the longer term though. A. Pielen et al. investigated the effect of switching to Dexamethasone implants in RVO-affected eyes refractory to anti-VEGF injections, previous Dexamethasone implants, or treatment-naive eyes. The results displayed herein were inconsistent: despite a significant reduction when switching from anti-VEGF to Dexamethasone, a significant improvement in BCVA failed. Overall, there were three more puzzle pieces of the signification of switching (anti-VEGF) drugs in the treatment strategy of neovascular eye disorders. The data precisely show that further research is essential to attain the best treatment option for the individual patient.

Y. Subhi and T. L. Sørensen investigated different aspects of nAMD patients older than 90 years of age and showed that overall 7% of nAMD patients treated belong to this group. In this subpopulation, treatment is oftentimes discontinued by death or various treatment burdens. Aflibercept showed superior visual as well as anatomical outcomes in comparison to Ranibizumab after 2 years. The authors conclude that new strategies regarding treatment burdens and the use of specific anti-VEGF substances might be needed for the future, as patients are getting older and older.

Beside efficacy, possible side effects of any anti-VEGF therapy emerged into the focus of vitreoretinal research. Specifically, the progression rate of retinal pigment epithelium (RPE) loss during intravitreal anti-VEGF treatment is an important aspect, because the induction of RPE atrophy might hamper visual recovery. J. Wons et al. compared the atrophy progression rates between Ranibizumab and Aflibercept in eyes suffering from nAMD and could show that no significant differences between both treatment modalities exist.

Another focus of this special issue turned out to be the effect of anti-VEGF therapy on eyes with retinopathy of prematurity (ROP). Since the ongoing RAINBOW extension study (ClinicalTrials.gov Identifier: NCT02640664) is evaluating the effect of Ranibizumab on functional and anatomical outcomes in ROP-affected eyes in a large, worldwide, clinical trial, this might become an approved therapeutic treatment option in the future and is therefore of immanent interest. In this regard, Q. Huang et al. underlined with their results published herein that eyes of infants treated bilaterally with intravitreally injected Ranibizumab can react differently and that reactivation after previous injections in ROP-affected eyes is an important issue ophthalmologists should be aware of. Furthermore, J. J. Tan et al. showed in a neonatal rat model that exposure of intermittent hypoxia-induced injured retinal microvasculature to anti-VEGF substances can result in vascular leakage and adverse effects in the developing neonate. This aspect is of clinical importance, because anti-VEGF use in the treatment of ROP infants is increasing by the day and caution regarding systemic side effects is warranted.

Beside these traditional indications for anti-VEGF treatment, new fields have arisen, among these was (neovascular) glaucoma. In many cases, especially in neovascular glaucoma, therapeutic approaches can be challenging. J. Kwon and K. R. Sung showed in their retrospective report of eyes suffering from neovascular glaucoma that preoperatively injected Bevacizumab before Ahmed glaucoma valve implantation can enhance overall success rates but conclude that subsequent prospective studies are needed to confirm this possible beneficial effect. M. Slabaugh and S. Salim not only report in their nicely written overview on the use of anti-VEGF substances in glaucoma surgery the potential benefit of these drugs but also claim that a precise role needs to be defined in the future.

Beside anti-VEGF agents, a bunch of new substances treating neovascular eye disorders are being developed and brought into phase I to III clinical trials. Before administered in first-in-man investigations, experimental approaches are needed to evaluate the potential efficacy and side effects. In this regard, C. Ren et al. reported an oral tyrosine kinase inhibitor, CM082, to treat experimental choroidal neovascularizations in rats. They could show that CM082 passed the blood-retina barrier and was detectable within the eyes in a reasonable concentration which in turn leads to significantly less neovascularization in comparison to controls. Thus, the idea of an oral application of drugs for the treatment of neovascular eye disorders seems to be viable. The future will tell if this molecule will find its way into clinical trials. Finally, D. Ning et al. demonstrated a novel pathway (Wnt/beta-catenin/COX-2/VEGF) to play a pathogenetic role in the development of retinopathies, and thus, this novel pathway might be a new target for future therapeutic approaches.

Overall, this special issue provides a variety of up-to-date basic and clinical research results of anti-VEGF and other substances targeting neovascular eye disorders. In light of the fact that different anti-PDGF molecules that were deemed to be the next step in the treatment of neovascular eye diseases and especially of nAMD-affected eyes recently failed in phase III clinical trials, it seems obvious that further intensive research is necessary to improve overall treatment outcomes for our patients.

